# Supplementary material for: Repurposing of Drugs as Novel Influenza Inhibitors From Clinical Gene Expression Infection Signatures
Source: Front Immunol. 2019 Jan 29;10:60. doi: 10.3389/fimmu.2019.00060 (PMC6361841; doi:10.3389/fimmu.2019.00060)
Supplement: Supplementary file 1 [file Data_Sheet_1.docx]

*Supplementary Material*

Repurposing of drugs as novel influenza inhibitors from clinical gene expression infection signatures

**Authors:** Andrés Pizzorno^¶^, Olivier Terrier^¶*^, Claire Nicolas de Lamballerie, Thomas Julien, Blandine Padey, Aurélien Traversier, Magali Roche, Marie-Eve Hamelin, Chantal Rhéaume, Séverine Croze, Vanessa Escuret, Julien Poissy^7^, Bruno Lina, Catherine Legras-Lachuer, Julien Textoris, Guy Boivin, Manuel Rosa-Calatrava^*^.

^*^ Corresponding authors:

[olivier.terrier@univ-lyon1.fr](mailto:olivier.terrier@univ-lyon1.fr) (OT), [manuel.rosa-calatrava@univ-lyon1.fr](mailto:manuel.rosa-calatrava@univ-lyon1.fr) (MRC)

**
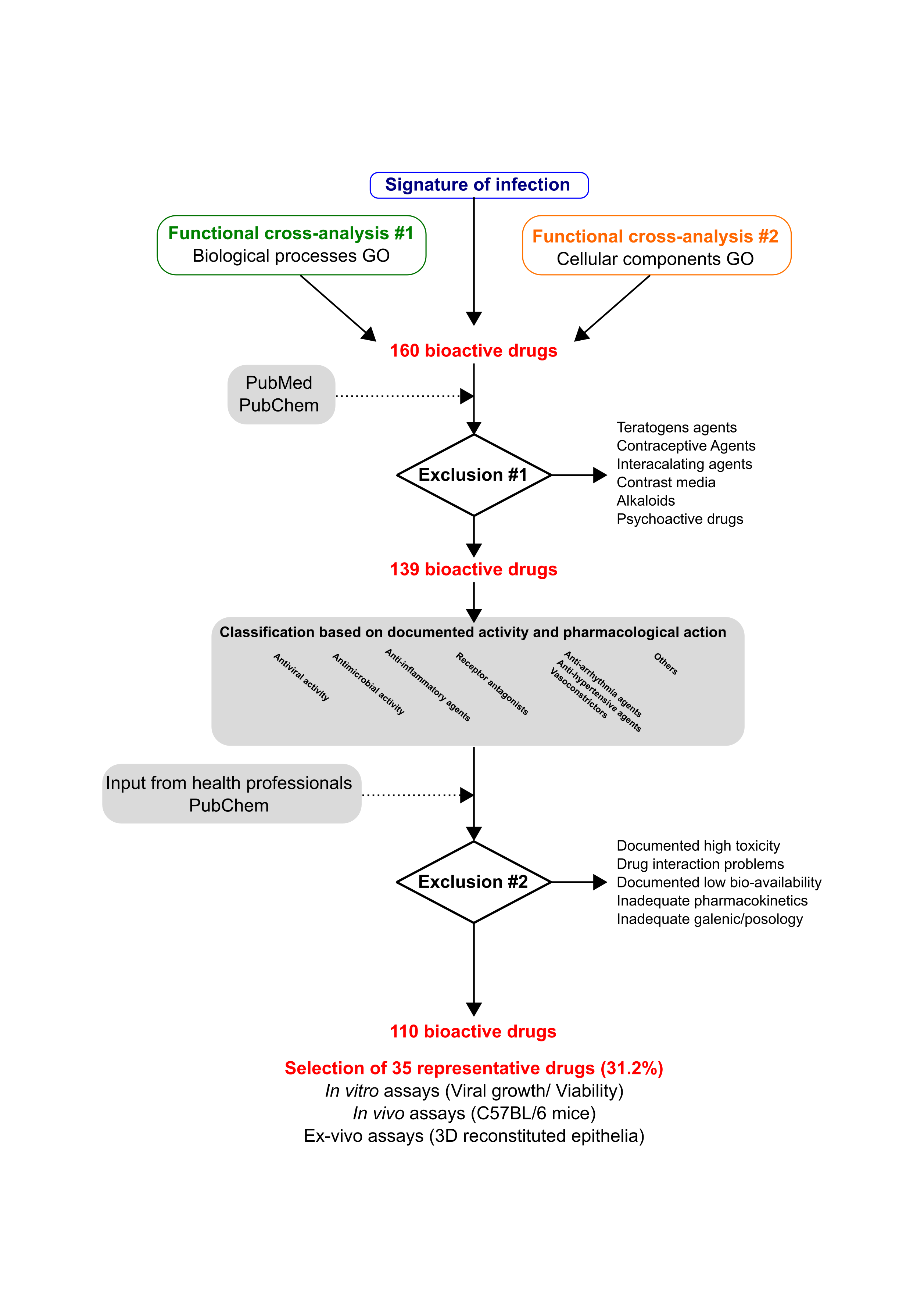
**

**Fig. S1. Decision tree used to rationally reduce the number of drug candidates.** Bioactive molecules were excluded if not compatible with a final use as antiviral, mostly for safety (e.g. teratogens, intercalating agents) and/or pharmacological (e.g. documented low bioavailability) reasons. An additional selection level based on analysis of documented pharmacological actions was included, to finally define a shortlist of 35 representative molecules (˂3% of CMAP) for in vitro screening (Table 1).

**
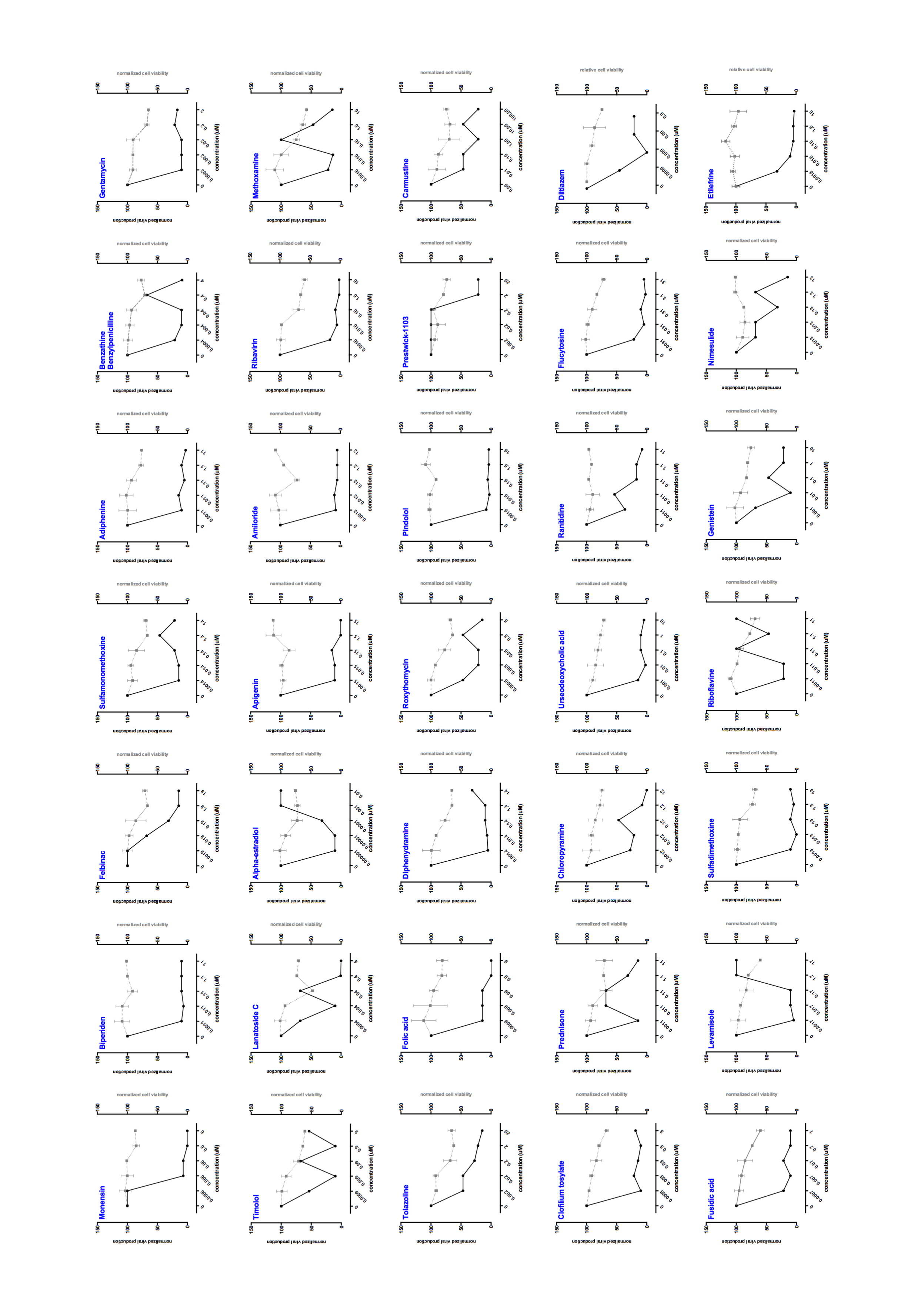
**

**Fig. S2. Dose-response curves for the 35 molecules tested *in vitro*.** In comparison with a mock-treated control, the impact of pre-treatment/treatment on % relative viral production (black line, left Y axis) and % relative cell viability (grey line, right Y axis) was measured at the indicated concentrations.

**
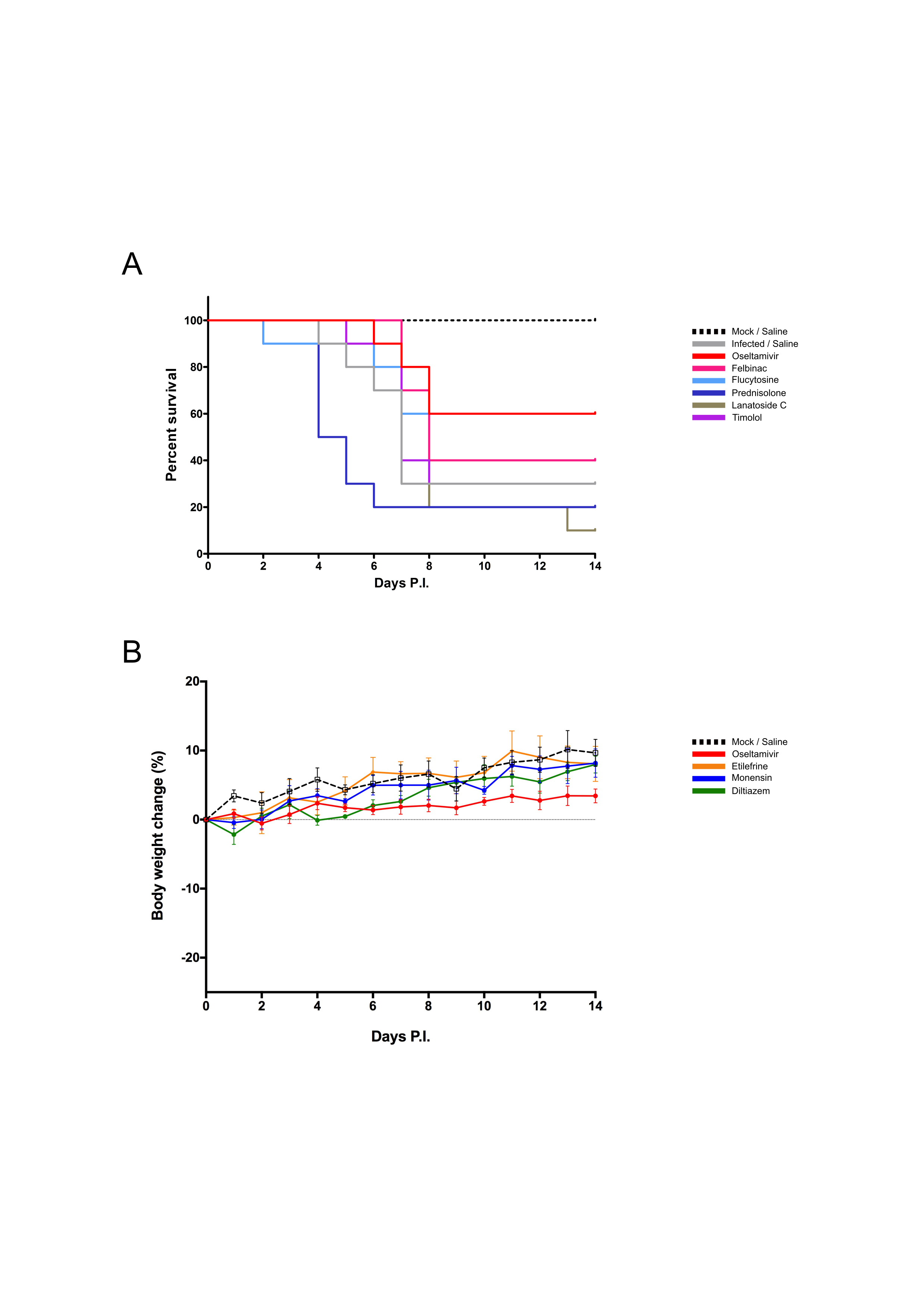
**

**Fig. S3. Efficacy and toxicity after oral administration of selected molecules in mice.** (A) Survival curves of C57BL/6N mice (n=15/group), intranasally inoculated with 5 x 10^5^ PFU of influenza A/Quebec/144147/09 virus on day 0 and treated by gavage with saline (grey), oseltamivir 10 mg/kg/day (red), lanatoside C 100 mg/kg/day (olive), prednisolone 5 mg/kg/day (dark blue), flucytosine 240 mg/kg/day (light blue), felbinac 5 mg/kg/day (fuchsia) or timolol 50 mg/kg/day (purple). A mock-infected, saline-treated group (black dotted line, n=6) was included as control. Treatments were initiated on day 0 (6 h before infection) and administered once daily for 5 consecutive days. (B) Body weight changes of mock-infected C57BL/6N mice (n=10/group) treated by gavage with saline (grey), oseltamivir 10 mg/kg/day (red), monensin 10 mg/kg/day (blue), diltiazem 90 mg/kg/day (green), or etilefrine 3 mg/kg/day (orange). A saline-treated group (black dotted line, n=6) was included as control. Treatments were initiated on day 0 (6 h before mock-infection) and administered once daily for 5 consecutive days.


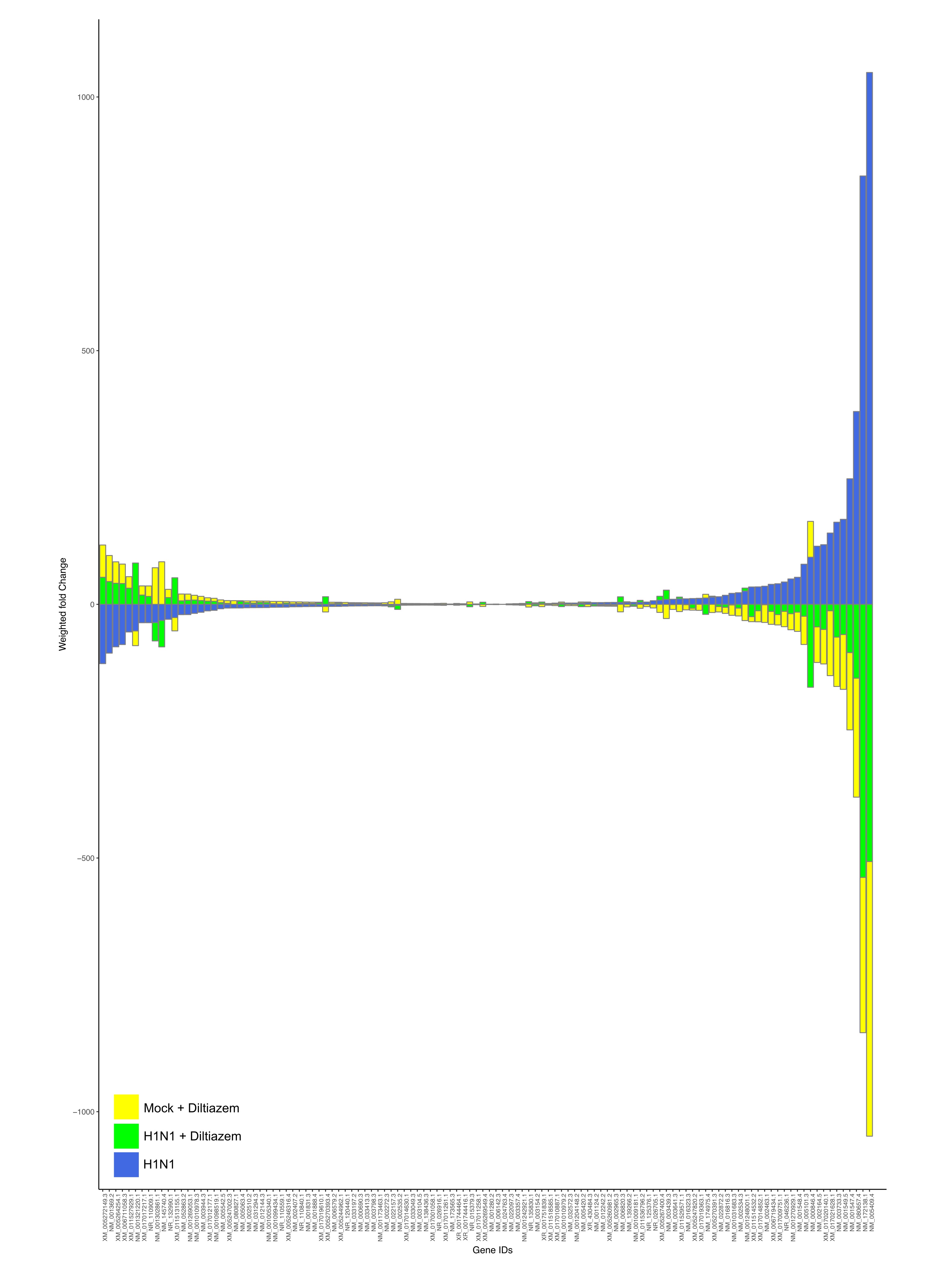


**Fig. S4.** **Diltiazem treatment effectively induces significant reversion of the viral infection signature.** Stacked barplots with mean-weighted fold changes for the complete list of the 118 common differentially expressed transcripts (absolute fold change >2, Benjamini-Hochberg corrected p-value <0.05) between the mock + diltiazem (yellow), H1N1 (blue), and H1N1 + diltiazem (green) conditions. Barplots were constructed in R3.3.1 based on mean-weighted fold changes and ordered according to H1N1 values (blue).

**
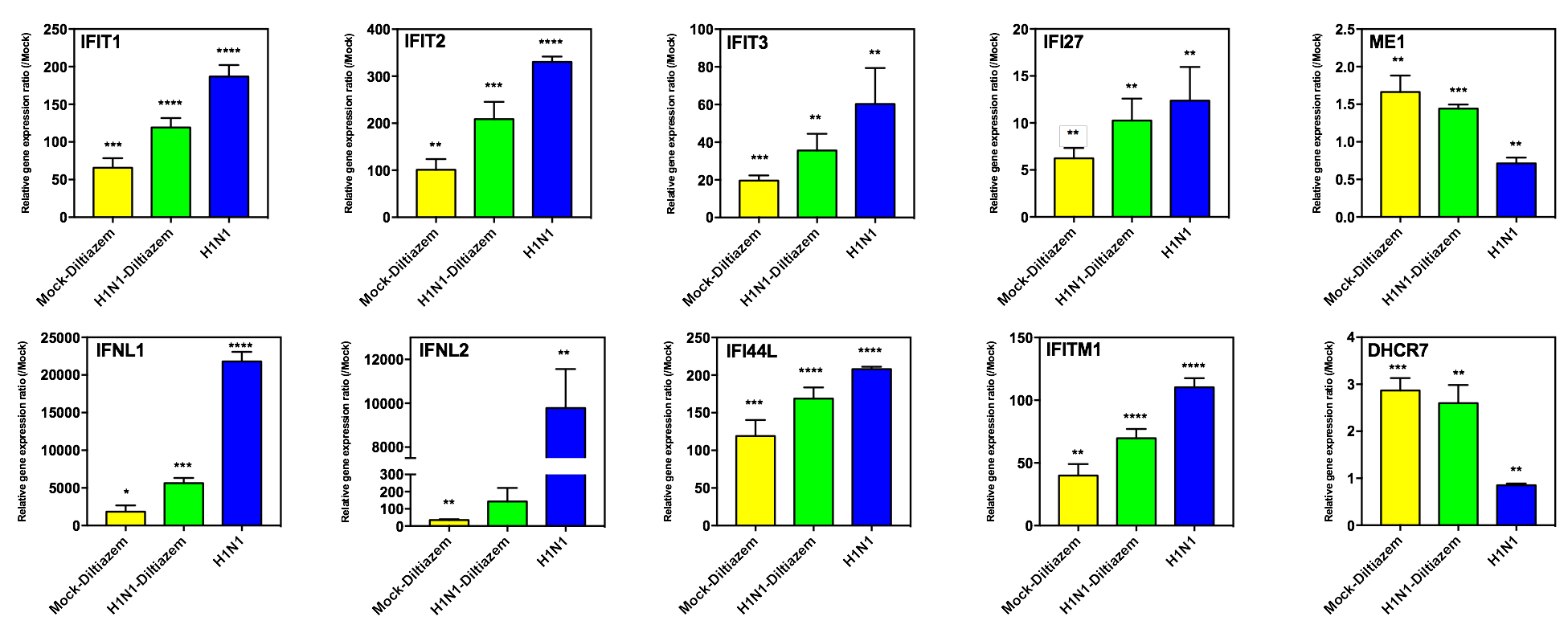
Fig. S5. RT-qPCR mRNA quantification performed on a set of genes related to the host antiviral response and cholesterol metabolism**. The relative mRNA level of 10 representative IFN-related or cholesterol-related genes was quantified for the same HAE infection / treatment conditions described in (A) and expressed as the mean fold change (±SD) compared to the mock condition. *P<0.05, **P<0.01, ***P<0.001 and ****P<0.0001 compared to the mock condition by unpaired t-test. Data are representative of at least two independent experiments.

| **Demographic Characteristics** |  |
| --- | --- |
| Number of patients | 9 |
| Male sex ratio (n,%) | 1 (11.1) |
| Age (Mean, SD) | 31.67 (8.9) |
| Min-Max age (years) | 19-42 |
| **Delay from beginning of symptoms** |  |
| Delay (Mean, SD) | 24.56 (8.8) |
| **Clinical symptoms** |  |
| Highest body Temperature (°C, mean, SD) | 38.98 (0.4) |
| **Constitutional symptoms** | |
| Chills and/or sweats (n,%) | 9 (100) |
| Aches (n,%) | 9 (100) |
| Fatigue (n,%) | 9 (100) |
| Headache (n,%) | 8 (88.9) |
| **Respiratory symptoms** | |
| Cough (n,%) | 7 (77.8) |
| Pharyngitis (n,%) | 3 (33.3) |
| Sore throat or nasal congestion (n,%) | 6 (66.7) |
| **Other symptoms** | |
| Expectoration (n,%) | 1 (11.1) |
| Otitis (n,%) | 0 (0.0) |
| Digestive disturbance (n,%) | 0 (0.0) |

**Table S1. Demographic and clinical characteristics of patients included in the study.** Samples were obtained in the context of a previous clinical trial conducted in France during the A(H1N1)pdm09 pandemic, aimed at evaluating the antiviral efficacy and tolerability of classic antiviral monotherapy versus bitherapy (Escuret et al., 2012).

| **Molecules** | **Pharmalogical class** |
| --- | --- |
| **Monensin*** | Antifungal Agents/Antiprotozoal Agents/Coccidiostats/Proton Ionophores/Sodium Ionophores |
| **Isoxicam** | Anti-Inflammatory Agents, Non-Steroidal |
| **Iloprost*** | Platelet Aggregation Inhibitors/Vasodilator Agents |
| **Securinine** | Alkaloids/GABAA Receptor Antagonists |
| **Prestwick-692** | Steroid Alkaloids |
| **Biperiden*** | Antiparkinson Agents/Muscarinic Antagonists/Parasympatholytics |
| **Clorsulon** | Anthelmintics/Antiplatyhelmintic Agents |
| **Felbinac** | Anti-Inflammatory Agents, Non-Steroidal |
| **Chenodeoxycholic acid*** | Cathartics/Gastrointestinal Agents |
| **Sulfamonomethoxine** | Anti-Infective Agents |
| **3-acetamidocoumarin** | ? |
| **Meteneprost** | Abortifacient Agents, Nonsteroidal |
| **Adiphenine** | Parasympatholytics/Anticholinergics/Antispamodics |
| **Benzathine benzylpenicillin** | Anti-Bacterial Agents |
| **Gentamicin** | Anti-Bacterial Agents/Protein Synthesis Inhibitors |
| **Timolol*** | Adrenergic beta-Antagonists/Anti-Arrhythmia Agents/Antihypertensive Agents |
| **Atractyloside*** | Enzyme Inhibitors |
| **Nadolol** | Adrenergic beta-Antagonists/Anti-Arrhythmia Agents/Antihypertensive Agents/Sympatholytics |
| **Lycorine*** | Alkaloids/Protein Synthesis Inhibitors |
| **Tranexamic acid*** | Antifibrinolytic Agents |
| **Lanatoside C** | Anti-Arrhythmia Agents |
| **Podophyllotoxin*** | Antineoplastic Agents, Phytogenic/Keratolytic Agents/Tubulin Modulators |
| **Alpha-estradiol*** | 5alpha-reductase inhibitors/Androgenic alopecia treatment |
| **Alprostadil** | Fibrinolytic Agents/Platelet Aggregation Inhibitors/Vasodilator Agents |
| **Apigenin*** | Anti-Inflammatory Agents, Non-Steroidal/? |
| **Pipenzolate bromide** | Muscarinic Receptor Antagonists |
| **Amiloride*** | Epithelial Sodium Channel Blockers/Diuretics/Acid Sensing Ion Channel Blockers |
| **STOCK1N-35696** | ? |
| **Carmustine** | Antineoplastic Agents, Alkylating |
| **PF-00539745-00** | ? |
| **Clofilium tosylate** | Anti-Arrhythmia Agents |
| **Prestwick-983*** | Antimetabolites/Antiviral Agents |
| **Prestwick-675** | Anthelmintics/Anticestodal Agents/Antiprotozoal Agents/Tubulin Modulators |
| **Atracurium besilate** | Neuromuscular Nondepolarizing Agents/Nicotinic Antagonists |
| **Xamoterol** | Adrenergic beta-1 Receptor Agonists |
| **Demecarium bromide** | Cholinesterase Inhibitors |
| **Iopromide** | Contrast Media |
| **Etilefrine** | Adrenergic alpha-Agonists/Adrenergic beta-1 Receptor Agonists/Cardiotonic Agents/Sympathomimetics/Vasoconstrictor Agents |
| **Iproniazid** | Antidepressive Agents/Monoamine Oxidase Inhibitors |
| **Ketotifen*** | Anti-Allergic Agents/Antipruritics/Histamine H1 Antagonists |
| **Gly-His-Lys** | Anti-Inflammatory Agents, Non-Steroidal? |
| **Canadine*** | Anti-Arrhythmia Agents/Calcium Channel Blockers/Platelet Aggregation Inhibitors |
| **Viomycin** | Anti-Bacterial Agents/Antibiotics, Antitubercular/Protein Synthesis Inhibitors |
| **Disopyramide** | Anti-Arrhythmia Agents/Voltage-Gated Sodium Channel Blockers |
| **Fusidic acid** | Anti-Bacterial Agents/Protein Synthesis Inhibitors |
| **Amiprilose** | Adjuvants, Immunologic/Anti-Inflammatory Agents, Non-Steroidal/Antiviral Agents |
| **Anisomycin*** | Anti-Bacterial Agents/Antiprotozoal Agents/Nucleic Acid Synthesis Inhibitors/Protein Synthesis Inhibitors |
| **Ajmaline** | Anti-Arrhythmia Agents |
| **Arecoline** | Cholinergic Agonists |
| **Metampicillin** | Anti-Bacterial Agents |
| **Lasalocid** | Anti-Bacterial Agents/Coccidiostats/Ionophores |
| **Tolnaftate** | Antifungal Agents |
| **Metixene** | Muscarinic Receptor Antagonists |
| **PF-00539758-00** | ? |
| **Streptomycin** | Anti-Bacterial Agents/Protein Synthesis Inhibitors |
| **Sulfapyridine** | Anti-Infective Agents/Dermatologic Agents |
| **Pivmecillinam** | Anti-Bacterial Agents/Anti-Infective Agents, Urinary |
| **Ribavirin*** | Antimetabolites/Antiviral Agents |
| **Prestwick-642*** | Dermatologic Agents/Teratogens |
| **Bumetanide** | Diuretics/Sodium Potassium Chloride Symporter Inhibitors |
| **Prednisone** | Anti-Inflammatory Agents/Antineoplastic Agents, Hormonal/Glucocorticoids |
| **Doxylamine** | Antiemetics/Histamine H1 Antagonists |
| **Diphenylpyraline** | Histamine H1 Antagonists |
| **Finasteride** | 5-alpha Reductase Inhibitors |
| **Rosiglitazone*** | Hypoglycemic Agents |
| **15-delta prostaglandin J2** | Anti-Inflammatory Agents, Non-Steroidal?/NFkB inhibitor? |
| **5230742** | ? |
| **Chloropyramine** | Histamine H1 Antagonists |
| **Prestwick-685** | Anti-Inflammatory Agents, Non-Steroidal/Coloring Agents/Leprostatic Agents |
| **Nimesulide*** | Anti-Inflammatory Agents, Non-Steroidal/Cyclooxygenase Inhibitors |
| **Morantel** | Anthelmintics/Antinematodal Agents |
| **Tropicamide** | Muscarinic Receptor Antagonists/Mydriatics |
| **Piperidolate** | Muscarinic Receptor Antagonists |
| **Riboflavin*** | Photosensitizing Agents/Vitamin B Complex |
| **Methoxamine** | Adrenergic alpha-1 Receptor Agonists/Sympathomimetics/Vasoconstrictor Agents |
| **Hydrocotarnine** | Alkaloids/? |
| **Propidium iodide** | Coloring Agents/Indicators and Reagents/Intercalating Agents |
| **Tolazoline** | Adrenergic alpha-Antagonists/Antihypertensive Agents/Vasodilator Agents |
| **5279552** | ? |
| **Sulfadimethoxine** | Anti-Infective Agents |
| **N-acetyl-L-leucine** | *Vertigo treatment/?* |
| **Gibberellic acid** | Plant Growth Regulators |
| **Clorgiline** | Antidepressive Agents/Monoamine Oxidase Inhibitors |
| **Genistein** | Anticarcinogenic Agents/Phytoestrogens/Protein Kinase Inhibitors |
| **Pargyline** | Antihypertensive Agents/Monoamine Oxidase Inhibitors |
| **Cortisone** | Anti-Inflammatory Agents |
| **Medrysone** | Anti-inflammatory Agents/Glucocorticoids |
| **Isoflupredone** | Anti-inflammatory Agents/Mineralcorticoids |
| **Prestwick-1082** | ? |
| **Aciclovir*** | Antiviral Agents |
| **Sulconazole** | Antifungal Agents |
| **Cycloserine** | Anti-Infective Agents, Urinary/Antibiotics, Antitubercular/Antimetabolites |
| **Procainamide** | Anti-Arrhythmia Agents/Voltage-Gated Sodium Channel Blockers |
| **Chlortalidone** | Antihypertensive Agents/Diuretics/Sodium Chloride Symporter Inhibitors |
| **Chlorzoxazone** | Muscle Relaxants, Central |
| **Oxolamine** | Antitussive Agents |
| **Folic acid** | Hematinics/Vitamin B Complex |
| **Furazolidone** | Anti-Infective Agents, Local/Anti-Infective Agents, Urinary/Antitrichomonal Agents/Monoamine Oxidase Inhibitors |
| **Cotinine** | Indicators and Reagents |
| **Ikarugamycin*** | Anti-Infective Agents |
| **H-7** | Enzyme Inhibitors |
| **Natamycin** | Anti-Bacterial Agents/Anti-Infective Agents, Local/Antifungal Agents |
| **H-89*** | Protein Kinase Inhibitors |
| **Guanadrel** | Antihypertensive Agents |
| **Midodrine** | Adrenergic alpha-1 Receptor Agonists/Sympathomimetics/Vasoconstrictor Agents |
| **Etiocholanolone** | Ketosteroids |
| **Methyldopate** | Antihypertensive Agents |
| **Oxymetazoline*** | Adrenergic alpha-Agonists/Nasal Decongestants/Sympathomimetics |
| **Levomepromazine** | Analgesics, Non-Narcotic/Antipsychotic Agents/Dopamine Antagonists |
| **Thapsigargin** | Enzyme Inhibitors |
| **Pyrithyldione** | Psychoactive drugs |
| **Nicergoline** | Adrenergic alpha-Antagonists/Nootropic Agents/Vasodilator Agents |
| **Apramycin** | Anti-Bacterial Agents |
| **Prestwick-1103** | Anti-Inflammatory Agents, Non-Steroidal/Cyclooxygenase Inhibitors |
| **Fenoprofen** | Anti-Inflammatory Agents, Non-Steroidal/Cyclooxygenase Inhibitors |
| **Fludrocortisone** | Mineralocorticoids/Anti-Inflammatory Agents |
| **Diphenhydramine** | Anesthetics, Local/Anti-Allergic Agents/Antiemetics/Histamine H1 Antagonists/Hypnotics and Sedatives |
| **Naloxone** | Narcotic Antagonists |
| **Benzonatate** | Antitussive Agents |
| **Thiocolchicoside** | Muscle Relaxants |
| **Eucatropine** | Mydriatics |
| **Dextromethorphan** | Antitussive Agents/Excitatory Amino Acid Antagonists |
| **Isometheptene** | Adrenergic alpha-1 Receptor Agonists/Sympathomimetics/Vasoconstrictor Agents |
| **Cinoxacin** | Anti-Infective Agents |
| **Levamisole*** | Adjuvants, Immunologic/Antinematodal Agents/Antirheumatic Agents |
| **Ursodeoxycholic acid** | Cholagogues and Choleretics |
| **4,5-dianilinophthalimide** | Protein Kinase Inhibitors |
| **Ifenprodil** | Adrenergic alpha-Antagonists/Excitatory Amino Acid Antagonists/Vasodilator Agents |
| **CP-320650-01** | ? |
| **Roxithromycin** | Anti-Bacterial Agents |
| **Lisuride** | Antiparkinson Agents/Dopamine Agonists/Serotonin Receptor Agonists |
| **Iomefloxacin** | Anti-Infective Agents |
| **Iorglumide** | Hormone Antagonists |
| **Piretanide** | Diuretics/Sodium Potassium Chloride Symporter Inhibitors |
| **L-methionine sulfoximine*** | Enzyme Inhibitors |
| **Diltiazem** | Antihypertensive Agents/Calcium Channel Blockers/Cardiovascular Agents/Vasodilator Agents |
| **Tyloxapol** | Detergents/Surface-Active Agents |
| **Flumequine** | Anti-Infective Agents/Anti-Infective Agents, Urinary |
| **Terazosin** | Adrenergic alpha-1 Receptor Antagonists |
| **Triflusal** | Platelet Aggregation Inhibitors |
| **Ranitidine** | Anti-Ulcer Agents/Histamine H2 Antagonists |
| **Flucytosine*** | Antifungal Agents/Antimetabolites |
| **Etomidate** | Anesthetics, Intravenous/Hypnotics and Sedatives |
| **Dioxybenzone** | *UVB/UVA protection?* |
| **Furaltadone** | Anti-Infective Agents, Urinary |
| **Ornidazole** | Amebicides/Antitrichomonal Agents/Radiation-Sensitizing Agents |
| **Dicloxacillin** | Anti-Bacterial Agents |
| **Pindolol** | Adrenergic beta-Antagonists/Antihypertensive Agents/Serotonin Antagonists/Vasodilator Agents |
| **Tretinoin*** | Antineoplastic Agents/Keratolytic Agents |
| **Proscillaridin** | Cardiotonic Agents/Enzyme Inhibitors |
| **Ouabain*** | Cardiotonic Agents/Enzyme Inhibitors |
| **Beclometasone** | Anti-Asthmatic Agents/Anti-Inflammatory Agents/Glucocorticoids |
| **Mexiletine** | Anti-Arrhythmia Agents/Voltage-Gated Sodium Channel Blockers |
| **Buflomedil** | Vasodilator Agents |
| **Levobunolol** | Adrenergic beta-Antagonists/Sympatholytics |
| **PHA-00851261E** | ? |
| **Estropipate** | Contraceptive Agents |
| **Ioversol** | Contrast Media |
| **0175029-0000** | ? |
| **Gelsemine** | Alkaloids/? |

**Table S2. List of 160 selected molecules and their documented pharmacological classes.** The 35 selected compounds for *in vitro* and *in vivo* evaluation are highlighted in grey. Asterisks (*) indicate molecules previously evaluated for their antiviral properties against influenza viruses or other viruses according to the literature, and question marks (?) indicate absence of assigned pharmacological class. Documented pharmacological classes were obtained from PubChem (<https://pubchem.ncbi.nlm.nih.gov)>.

| **Molecule** | **CMAP (µM)** | **EC50 (nM)** |
| --- | --- | --- |
| Monensin | 6 | 3.27 |
| Biperiden | 11 | 0.38 |
| Felbinac | 19 | 34.35 |
| Sulfamonomethoxine | 14 | 0.28 |
| Adiphenine | 11 | 6.29 |
| Benzathin Benzylpenicilline | 4 | 0.26 |
| Gentamycin | 3 | 0.039 |
| Timolol | 9 | nd |
| Lanatoside C | 4 | nd |
| Alpha-estradiol | 0.01 | nd |
| Apigenin | 15 | 0.21 |
| Amiloride | 13 | 0.41 |
| Ribavirin | 16 | 0.86 |
| Methoxamine | 16 | nd |
| Tolazoline | 20 | 0.93 |
| Folic acid | 9 | 0.22 |
| Diphenydramine | 14 | 0.25 |
| Roxythromycin | 5 | 0.14 |
| Pindolol | 16 | 0.79 |
| Prestwick-1103 | 20 | 626.63 |
| Carmustine | 100 | 0.07 |
| Clofilium tosylate | 8 | 0.16 |
| Prednisone | 11 | nd |
| Choropyramine | 12 | 0.07 |
| Urseodeoxycholic acid | 10 | 0.61 |
| Ranitidine | 11 | 0.68 |
| Flucytosine | 31 | 2.02 |
| Diltiazem | 9 | 0.84 |
| Fusidic acid | 7 | 0.038 |
| Levamisole | 17 | nd |
| Sulfadimethoxine | 13 | 0.69 |
| Riboflavine | 11 | nd |
| Genistein | 10 | 1.06 |
| Nimesulide | 13 | nd |
| Etilefrine | 18 | 0.34 |

**Table S3. List of 35 selected molecules.** CMAP concentration (µM) and calculated EC50 in the context of pre-treatment/treatment *in vitro*.

| **Influenza virus** | **Treatment** | **Dose** | **Viral titer (log TCID50/ml)** | **Relative Viral production**  **(% of mock-treated)** |
| --- | --- | --- | --- | --- |
| A/Lyon/969/2009 H275Y (**H1N1**)  MOI 0.1 | Diltiazem | 0 | 4.8 | 100 |
|  |  | CMAP/10 | 3.97 | 14.7 |
|  |  | CMAP | 2.8 | 1.0 |
|  | Etilefrine | 0 | 4.63 | 100 |
|  |  | CMAP/10 | 3.97 | 22.0 |
|  |  | CMAP | 2.8 | 1.5 |
| A/Texas/126/2016 (**H3N2**)  MOI 0.01 | Diltiazem | 0 | 6.02 | 100 |
|  |  | CMAP/10 | 5.13 | 12.75 |
|  |  | CMAP | 4.92 | 7.9 |
|  | Etilefrine | 0 | 5.63 | 100 |
|  |  | CMAP/10 | 4.8 | 14.7 |
|  |  | CMAP | 3.63 | 1 |
| **B**/Massachusetts/2/2106  MOI 0.1 | Diltiazem | 0 | 5.3 | 100 |
|  |  | CMAP/10 | 4.3 | 10.0 |
|  |  | CMAP | 3.97 | 4.7 |
|  | Etilefrine | 0 | 5.13 | 100 |
|  |  | CMAP/10 | 4.3 | 15.0 |
|  |  | CMAP | 3.97 | 7.3 |

**Table S4.** **Evaluation of antiviral efficacy of diltiazem or etilefrine in the context of infection by different influenza strains.** Human lung epithelial cells (A549) were incubated with supplemented medium (mock), or different concentrations of diltiazem (CMAP, 9 µM) or etilefrine (CMAP, 18 µM). Six hours after treatment, cells were washed and then infected with different prototype human influenza strains (as indicated). One hour after viral infection, a second identical treatment dose in supplemented medium was added. Relative viral titers compared to the mock-treated control are shown. Results are representative of two independent experiments, and confirm the antiviral activity of diltiazem and etilefrine on oseltamivir-resistant A(H1N1)pdm09, as well as wild-type H3N2 and B influenza strains.

| **Pre-incubation treatment** | **Viral titer (log TCID50/ml)** | | **Mean relative Viral production**  **(% of mock-treated)** |
| --- | --- | --- | --- |
|  | **Dilution #1** | **Dilution #2** |  |
| **Control PBS** | 5.3 | 4.63 | - |
| **Diltiazem** (9 µM) | 5.63 | 4.63 | 156.9 |
| **Etilefrine** (18 µM) | 5.30 | 4.63 | 100.0 |
| **Oseltamivir** (1 µM) | 5.63 | 4.30 | 130.3 |
| **Negative serum** | 4.97 | 4.30 | 47.7 |
| **Positive serum** | 3.30 | 2.53 | 0.9 |

**Table S5. Virus pre-incubation with diltiazem or etilefrine does not interfere with early viral entry steps.** Two viral dilutions (#1 and #2, respectively 10^6^ and 10^5^ TCID50/mL) were pre-incubated for 1 h with PBS, diltiazem (CMAP, 9 µM), etilefrine (CMAP, 18 µM), or oseltamivir (1 µM). A(H1N1)pdm09 positive and a negative sera were used as controls. After incubation, viral titers (log10 TCID50/mL) were determined in MDCK cells. Results are representative of two independent experiments and indicate that pre-incubation with either diltiazem or etilefrine does not affect viral titers compared to PBS-incubated control, suggesting that the antiviral effect of these molecules is not mediated by direct drug-virus interactions at early stages of viral entry.

| **Hours P.I.** | **Treatment** | **Apical viral titer**  **PFU/ml**  **(CI95)** | **Apical viral titer**  **log TCID50/ml**  **(CI95)** | **Δ TEER**  **Ohm/cm^2^**  **(CI95)** |
| --- | --- | --- | --- | --- |
| **24** | Mock | **7.2^5** (2.3^5 – 1.2^6) | **6.74** (6.53 – 6.95) | **59.84** (-35.75 – 155.4) |
|  | Oseltamivir 0.1 µM | **2.1^5** (1.6^4 – 4.1^5) | **6.02** (4.69 – 7.35) | **8.98** (-81.72 – 99.68) |
|  | Oseltamivir 1 µM | **2.1^4***** (2.3^3 – 3.9^4) | **5.13***** (4.61 – 5.66) | **-24.32** (-58.00 – 9.36) |
|  | Diltiazem 9 µM | **3.8^6** (1.6^5 – 6.0^5) | **6.47** (5.03 – 7.90) | **35.10** (10.96 – 59.23) |
|  | Diltiazem 90 µM | **3.1^4***** (1.8^5 – 4.3^5) | **5.57**** (4.95 – 6.18) | **86.13** (3.72 – 168.5) |
|  | Ose 0.1 µM / Dil 9 µM | **5.3^4***** (1.7^3 – 1.0^5) | **6.25** (5.62 – 6.89) | **34.50** (-33.15 – 102.2) |
|  | Ose 1 µM / Dil 90 µM | **2.3^4***** (1.9^4 – 2.7^4) | **5.19***** (4.15 – 6.23) | **32.96** (-77.24 – 143.2) |
| **48** | Mock | **2.1^8** (1.3^8 – 3.0^8) | **9.12** (8.91 – 9.33) | **-2.64** (-233.3 – 228.0) |
|  | Oseltamivir 0.1 µM | **5.9^7**** (4.9^7 – 6.8^7) | **8.61** (7.43 – 9.80) | **20.09** (-39.75 – 79.94) |
|  | Oseltamivir 1 µM | **8.8^4***** (3.2^3 – 1.7^5) | **6.20***** (5.39 – 7.01) | **7.26** (-85.73 – 100.3) |
|  | Diltiazem 9 µM | **5.8^7**** (3.0^7 – 8.5^7) | **8.91** (8.67 – 9.16) | **-48.31** (-180.1 – 83.47) |
|  | Diltiazem 90 µM | **1.0^5***** (4.4^4 – 1.5^5) | **6.56***** (5.23 – 7.88) | **120.1** (-85.77 – 326.00) |
|  | Ose 0.1 µM / Dil 9 µM | **2.3^6***** (9.7^5 – 3.6^6) | **7.54*** (6.92 – 8.17) | **78.28** (33.37 – 123.2) |
|  | Ose 1 µM / Dil 90 µM | **2.6^4***** (1.4^4 – 3.8^4) | **5.38***** (5.05 – 5.71) | **-8-84** (-58.73 – 41.05) |
| **72** | Mock | **1.2^8** (3.3^7 – 2.1^8) | **8.48** (8.02 – 8.94) | **-244.1** (-275.2 – -213.0) |
|  | Oseltamivir 0.1 µM | **4.6^7*** (7.6^6 – 8.4^7) | **8.30** (7.73 – 8.87) | **-110.9*** (-256.0 – 34.2) |
|  | Oseltamivir 1 µM | **1.7^5***** (7.1^4 – 2.7^5) | **6.85***** (6.14 – 7.56) | **22.55***** (-20.25 – 65.35) |
|  | Diltiazem 9 µM | **1.9^7**** (-4.7^5 – 3.9^7) | **8.21** (7.28 – 9.14) | **-218.0** (255.6 – -180.4) |
|  | Diltiazem 90 µM | **2.1^5***** (1.3^5 – 3.0^5) | **7.38*** (6.53 – 8.22) | **-115.5** (-308.5 – 77.52) |
|  | Ose 0.1 µM / Dil 9 µM | **1.8^7**** (6.9^6 – 2.8^7) | **8.41** (7.38 – 9.45) | **-37.88**** (-215.3 – 139.6) |
|  | Ose 1 µM / Dil 90 µM | **4.7^4***** (3.8^4 – 5.7^4) | **5.62***** (5.23 – 6.01) | **-43.16**** (-77.17 – -9.15) |
| **96** | Mock | **2.8^7** (1.3^7 – 4.2^7) | **7.86** (7.74 – 7.99) | **-267.3** (-288.7 – -246.0) |
|  | Oseltamivir 0.1 µM | **4.1^6***** (-2.3^6 – 1.0^7) | **7.80** (6.56 – 9.04) | **-209.2** (-271.5 – -146.8) |
|  | Oseltamivir 1 µM | **1.8^5***** (6.3^3 – 3.4^5) | **7.09** (5.79 – 8.40) | **10.67***** (-27.47 – 48.81) |
|  | Diltiazem 9 µM | **2.3^6***** (1.9^6 – 2.7^6) | **7.32** (6.43 – 8.22) | **-220.5** (-267.4 – -173.5) |
|  | Diltiazem 90 µM | **1.6^5***** (-5.6^4 – 3.7^5) | **7.37** (5.97 – 8.76) | **24.59***** (-10.79 – 59.97) |
|  | Ose 0.1 µM / Dil 9 µM | **4.0^6***** (1.2^6 – 6.8^6) | **7.84** (7.38 – 8.31) | **-149.9*** (-379.8 – 80.1) |
|  | Ose 1 µM / Dil 90 µM | **2.9^4***** (2.1^4 – 3.7^4) | **5.47**** (4.38 – 6.56) | **-59.79***** (-97.62 – -21.96) |

**Table S6.** **Apical viral production and transepithelial electrical resistance (TEER) in infected MucilAir® human airway epithelium (HAE).** MucilAir® HAE were infected on the apical pole with influenza A/Lyon/969/09 (H1N1)pdm09 virus at a MOI of 0.1 and treated on the basolateral pole. Treatments were initiated 5 h after infection and were continued once daily for 4 additional days. *p<0.05, **p<0.01 and ***p<0.001 compared to the infected mock-treated group by one-way ANOVA with Tukey’s post-test. Data are representative of at least three independent experiments.
